# Potential Effect of Prolonged Sevoflurane Anesthesia on the Kinetics of [^11^C]Raclopride in Non-human Primates

**DOI:** 10.1007/s11307-017-1120-8

**Published:** 2017-09-15

**Authors:** Ryosuke Arakawa, Lars Farde, Junya Matsumoto, Naoki Kanegawa, Igor Yakushev, Kai-Chun Yang, Akihiro Takano

**Affiliations:** 1 Department of Clinical Neuroscience, Center for Psychiatry Research, Karolinska Institutet and Stockholm County Council, Stockholm, Sweden; 2 Personalized Health Care and Biomarkers, AstraZeneca PET Science Center, Karolinska Institutet, Stockholm, Sweden; 3 Department of Neuropsychiatry, School of Medicine, Fukushima Medical University, Fukushima, Japan; 4 Department of Nuclear Medicine and TUM Neuroimaging Center (TUM-NIC), Technische Universität München, Munich, Germany

**Keywords:** [^11^C]Raclopride, Binding potential, Positron emission tomography, Sevoflurane, Time-activity curve, Time to peak

## Abstract

**Purpose:**

Positron emission tomography (PET) in non-human primates (NHP) is commonly performed under anesthesia, with sevoflurane being a widely used inhaled anesthetic. PET measurement in NHP can be repeated, and a difference in radioligand kinetics has previously been observed between the first and second PET measurement on the same day using sevoflurane anesthesia. In this study, we evaluated the effect of prolonged sevoflurane anesthesia on kinetics and binding potential (BP_ND_) of [^11^C]raclopride in NHP.

**Procedures:**

Three cynomolgus monkeys underwent two to three PET measurements with [^11^C]raclopride under continuous sevoflurane anesthesia on the same day. The concentration of sevoflurane was adjusted according to the general conditions and safety parameters of the NHP. Time to peak (TTP) radioactivity in the striatum was estimated from time-activity curves (TACs). The BP_ND_ in the striatum was calculated by the simplified reference tissue model using the cerebellum as reference region.

**Results:**

In each NHP, the TTP became shorter in the later PET measurements than in the first one. Across all measurements (*n* = 8), concentration of sevoflurane correlated with TTP (Spearman’s *ρ* = − 0.79, *p* = 0.03), but not with BP_ND_ (*ρ* = − 0.25, *p* = 0.55).

**Conclusions:**

These data suggest that sevoflurane affects the shape of TACs but has no evident effect on BP_ND_ in consecutive PET measurements.

## Introduction

Positron emission tomography (PET) is widely used for examination of radioligand binding to receptors, enzymes, and transporters in the non-human primate (NHP) and human brain *in vivo* [1, 2]. A common approach is to use PET studies in NHP for the development of new radioligands, *i.e.*, to assess whether the radioligand provides sufficient brain exposure and appropriate kinetic behavior. Another common approach is to use NHP to facilitate drug development by demonstration of brain exposure by microdosing or occupancy at the intended drug target.

PET studies in NHP are most often performed under anesthesia to maintain the position of the head during time of data acquisition. Inhalational anesthesia such as sevoflurane, isoflurane, and halothane is the most often used method for NHP study. Especially sevoflurane is currently widely used.

PET measurement in NHP can typically be performed up to three times in one experimental day [3–5]. Such series of measurements allows for estimation of the blocking or displacement effect of drugs, release of neurotransmitters in different conditions, or assessment of the test-retest reproducibility of a PET radioligand, in a single day. For optimal comparisons, experimental conditions should be identical among serial PET measurements. However, the concentration of anesthesia is practically adjusted to the condition of the individual NHP, and may consequently vary over time.

Importantly, in several studies using sevoflurane anesthesia, a difference in radioligand kinetics has been observed between the first and second PET measurement on the same day. For example, [^11^C]*calbonyl*-raclopride and [^11^C]*methyl*-raclopride, which are the same structure except radio-labeling position, showed different brain kinetics with the shift of time to peak (TTP) between first and second PET [6]. Other studies showed the different brain kinetics in initial part between first (for baseline) and second (for displacement) PET measurements of [^11^C]AZ10419369 for serotonin 1B receptor [7] and [^11^C]T-773 for phosphodiesterase 10A (PDE10A) [8]. Two PET measurements of these studies were conducted serially in one day. Whereas an effect of sevoflurane anesthesia on cerebral blood flow [9–12], cerebral vasodilation [13], and brain metabolic rate [10, 12] has been reported, the effect of sevoflurane anesthesia on radioligand kinetics has not been investigated in detail.

In the present study, we evaluated the change of brain kinetics of [^11^C]raclopride, especially the shift of TTP, in relation to the concentration or duration of sevoflurane using repeated PET measurements in NHPs on the same day. In addition to the change of brain kinetics, we also estimated how altered brain kinetics may propagate into changes in the binding potential (BP_ND_) for [^11^C]raclopride in the striatum.

## Methods

### Subjects

Three cynomolgus monkeys (three females, body weight 4450, 4625, and 6065 g) were included in this study. The NHPs were housed in the Astrid Fagraeus Laboratory (AFL) of the Swedish Institute for Infectious Disease Control, Solna, Sweden. The study was approved by the Animal Ethics Committee of the Swedish Animal Welfare Agency and was performed according to “Guidelines for planning, conducting and documenting experimental research” (Dnr 4820/06-600) of Karolinska Institutet.

### PET Measurements

Anesthesia was induced by intramuscular injection of ketamine hydrochloride (approximately 10 mg/kg) at AFL, and then NHPs were transported to the Karolinska Institutet PET center. Inhalation anesthesia by administration of a mixture of sevoflurane, oxygen, and medical air started 40 min after ketamine injection and maintained with endotracheal intubation. The concentration of sevoflurane was adjusted by certified registered nurse anesthetists (CRNAs) according to the general condition of the NHP, mainly heart rate and blood pressure. The concentration of sevoflurane was recorded throughout the experiments.

The head was immobilized with a fixation device. Body temperature was maintained by a Bair Hugger model 505 (Arizant Healthcare, MN, USA) and monitored by an esophageal thermometer. ECG, heart rate, blood pressure, respiratory rate, and oxygen saturation were continuously monitored throughout the experiments. Fluid balance was maintained by a continuous infusion of saline.

PET measurements were conducted using the High Resolution Research Tomograph (HRRT) (Siemens Molecular Imaging, TN, USA) [14]. A transmission scan of 6 min using a single ^137^Cs source was performed before the emission scan. List mode data were acquired continuously for 63 min immediately after intravenous injection of [^11^C]raclopride, which was prepared by reported method [15]. The injected radioactivity (*n* = 8) was 153.1 ± 6.7 (mean ± SD) (range 146–165) MBq. NHP1 and NHP2 were examined three times, and NHP3 was examined twice in one experimental day.

### Data Analysis

The regions of interest (ROIs) were delineated manually on the MRI images of each NHP for striatum (combination of caudate and putamen) and cerebellum. The summed PET images of whole scanning were co-registered to the MRI image of the individual NHP. After applying the co-registration parameters to the dynamic PET data, the time-activity curves (TAC) of brain regions were generated for each PET measurement. BP_ND_ and R_1_ of the striatum were calculated by simplified reference tissue model (SRTM) [16] using the cerebellum as a reference region. The analyses of imaging data were performed using PMOD (version 3.6; PMOD Technologies, Zurich, Switzerland). To measure TTP of TAC in the striatum and cerebellum, a rational polynomial model was applied for noise reduction using MATLAB2013 (The Mathworks, MA, USA).

### Statistics

The relation between the concentration of sevoflurane and TTP of TAC in the striatum or cerebellum was assessed by Spearman’s rank correlation coefficient. The average concentration from − 15 to + 15 min of radioligand injection was used for the analysis. The relation between the concentration of sevoflurane and BP_ND_ or R_1_ in the striatum was also assessed by Spearman’s rank correlation coefficient. Additionally, the relation between the duration of sevoflurane anesthesia and TTP in the striatum or cerebellum, BP_ND_ or R_1_ was assessed by Spearman’s rank correlation coefficient. In all tests, a *p* value of < 0.05 (two-tailed) was considered statistically significant.

## Results

**Fig. 1.**
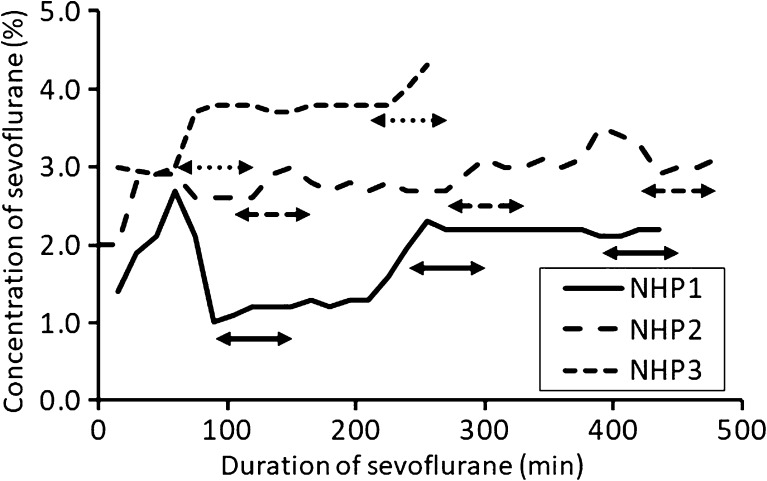
The time-courses of concentration of sevoflurane anesthesia in three NHPs. The arrows indicate the period of PET measurement.

All PET measurements were run according to the protocol. The concentration and duration of sevoflurane anesthesia, TTP of the TAC in the striatum and cerebellum, and BP_ND_ and R_1_ for all measurements are shown in Table 1. The concentration of sevoflurane increased with time (Fig. 1).

**Table 1:** The average concentration and duration of sevoflurane anesthesia, time to peak (TTP) of TAC in the striatum and cerebellum, and BP_ND_ and R_1_in the striatum

		PET1	PET2	PET3
NHP1	Conc	1.40	1.95	2.13
	Dur	111	254	400
	TTP_str	20.7	13.9	12.3
	TTP_cer	2.1	1.4	1.6
	BP_ND_	4.56	4.28	4.59
	R_1_	0.96	1.09	1.05
NHP2	Conc	2.60	2.77	3.20
	Dur	120	286	437
	TTP_str	13.2	8.3	6.2
	TTP_cer	1.6	1.7	1.3
	BP_ND_	4.55	4.56	4.93
	R_1_	0.99	1.09	1.30
NHP3	Conc	3.20	3.80	
	Dur	73	218	
	TTP_str	13.5	5.8	
	TTP_cer	1.3	1.3	
	BP_ND_	4.54	4.09	
	R_1_	0.85	0.98	

*Conc*, average concentration of sevoflurane anesthesia (%); *Dur*, duration of sevoflurane anesthesia (min); *TTP_str*, time to peak (TTP) of TAC in the striatum (min); *TTP_cer*, time to peak (TTP) of TAC in the cerebellum (min); *BP*_*ND*_, binding potential in the striatum; *R*_*1*_, R_1_ value in the striatum

**Fig. 2.**
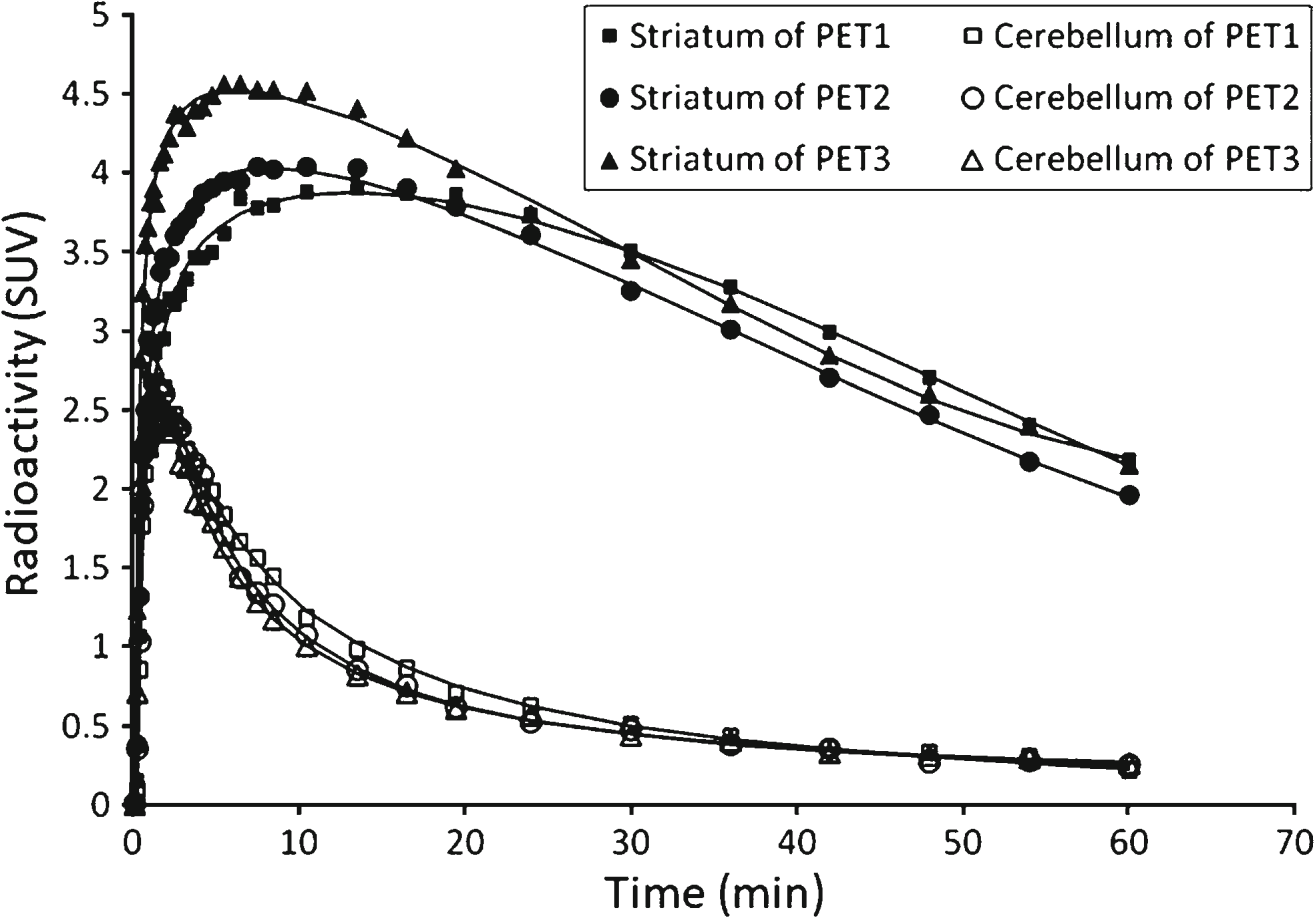
Typical time-activity curves (TACs) of NHP2.

**Fig. 3.**
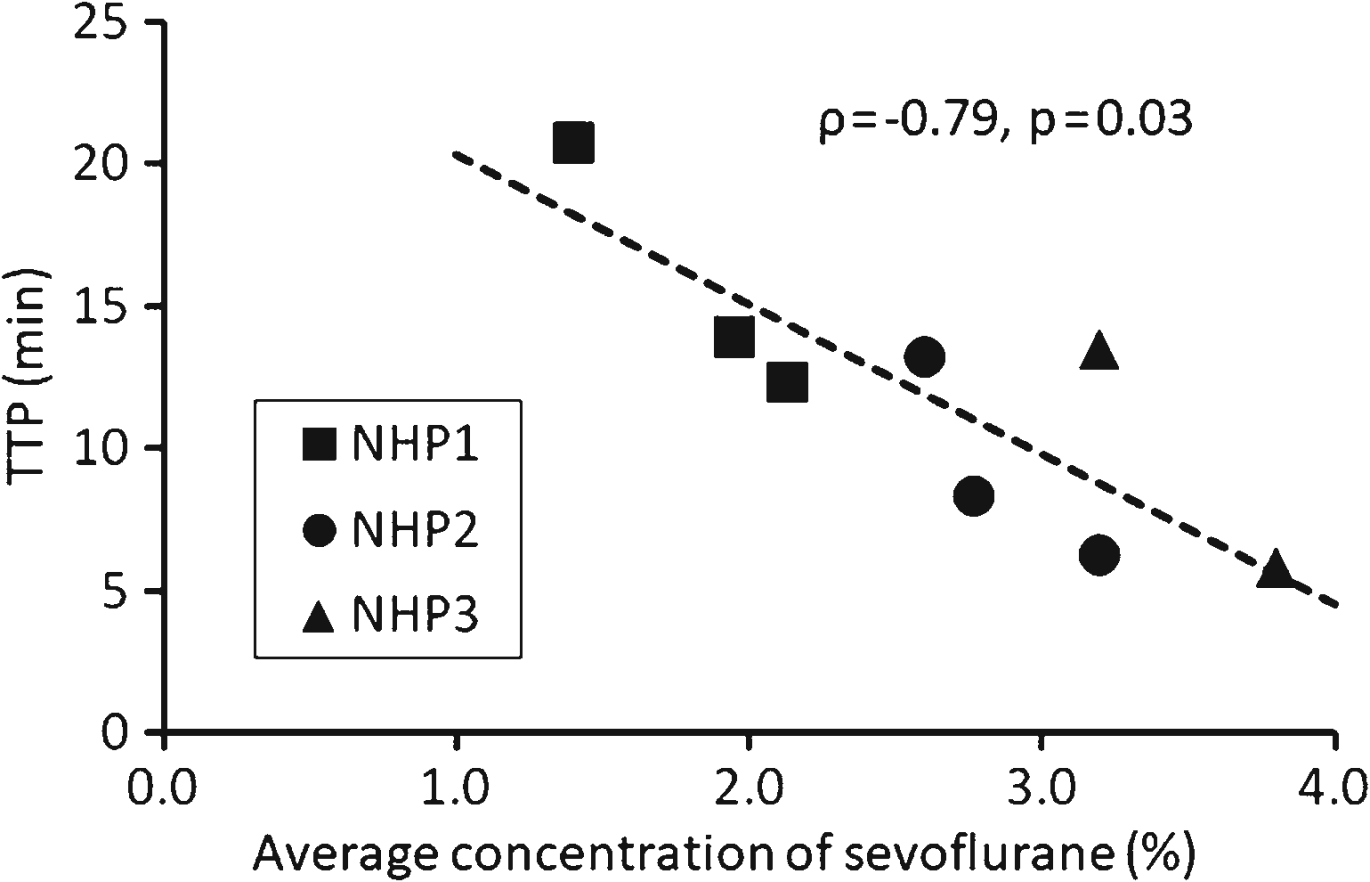
The relation between the average concentration of sevoflurane anesthesia and time to peak (TTP) of TAC in the striatum.

In all three NHPs, the TTP in the striatum became shorter in the later PET measurements than in the first one (Table 1, Fig. 2).When data for all three NHPs were combined, there was a statistically significant correlation (Spearman’s *ρ* = − 0.79, *p* = 0.03) between the concentration of sevoflurane and TTP of TAC in the striatum (Fig. 3). The correlation between the duration of sevoflurane anesthesia and TTP of TAC in the striatum was negative but not significant (*ρ* = − 0.57, *p* = 0.15). For TTP in the cerebellum, significant correlation was obtained for concentration (*ρ* = − 0.75, *p* = 0.04), but not duration (*ρ* = − 0.07, *p* = 0.87).

There was no obvious intra-individual difference between the BP_ND_ values obtained in each NHP. Moreover, there were no significant correlations between BP_ND_ and concentration (*ρ* = − 0.25, *p* = 0.55) or duration (*ρ* = 0.52, *p* = 0.20) of sevoflurane anesthesia. For R_1_, significant correlation was obtained for duration (*ρ* = 0.88, *p* = 0.01), but not concentration (*ρ* = − 0.06, *p* = 0.90).

## Discussion

In the present study, there was a significant negative correlation between the concentration of sevoflurane administered by inhalation and TTP of the brain TAC of [^11^C]raclopride. However, there was no significant correlation between the concentration of sevoflurane and binding potential of [^11^C]raclopride in the striatum.

The pharmacological characteristics of sevoflurane, such as the binding profile, are poorly described in the literature primarily since the binding of gases cannot be characterized in test tube experiments. Though the mechanism of action for sevoflurane is unclear, some functional effects may deserve attention. A change of cerebral blood flow (CBF) could be a possible reason for a change of brain kinetics, since the kinetic parameters across the blood-brain barrier, such as K_1_ in a compartment model, are dependent of CBF. In previous human studies, it has been reported that sevoflurane at about 1.0 minimum alveolar concentration (MAC) has an effect on CBF. However, the results are not consistent across the literature [9–13]. When the concentration of sevoflurane was higher than 1.5 MAC (approximately above 2 % inhaled), two studies [10, 13] have shown that sevoflurane increases CBF whereas another study [9] reported that sevoflurane decreases CBF. It is anyhow plausible that the relatively high concentration of sevoflurane in the present study may increase CBF, and induce the shift of TTP in radioligand kinetics.

Another factor which may change radioligand kinetics and the rate constant K_1_ is the blood-brain barrier (BBB) permeability. Some studies have indicated that sevoflurane has an effect on vascular endothelial cells thereby increasing brain permeability [17, 18]. Though this effect remains to be confirmed in intact tissue, it cannot be excluded that the more rapid brain kinetics observed at higher sevoflurane concentrations to some degree may be explained by a change in BBB permeability.

Despite the change in radioligand kinetics, there was no significant difference between the calculated BP_ND_ values of [^11^C]raclopride in consecutive PET measurements. Additionally, the BP_ND_ value did not correlate with the concentration of sevoflurane. TTP in the cerebellum became shorter in the latter PET measurements as well as the striatum. This shortening was correlated with sevoflurane concentration. The results suggested that the non-displaceable binding changed by anesthesia since cerebellum is devoid of specific binding. This change might contribute the change of the shape of TAC in the striatum. A small change of BP_ND_ induced by sevoflurane can anyhow not be excluded. [^11^C]Raclopride is sensitive to changes of endogenous dopamine, and sevoflurane has been reported to increase dopamine release in a microdialysis study in rodents [19]. However, the effect on dopamine release by sevoflurane (130–150 % of baseline) was much lower than the effect of methamphetamine (over 2000 % of baseline). Since it has been estimated that a 40 % increase in dopamine corresponds to a 1 % reduction of [^11^C]raclopride BP_ND_ [20, 21], a hypothetical change of BP_ND_ by sevoflurane should likely be negligible.

Some articles have reported regional difference in the effect of sevoflurane on CBF [9, 11]. The present study showed a significant correlation between duration of sevoflurane and R_1_. However, there was no significant correlation between concentration of sevoflurane and R_1_. These observations suggest that the prolonged effect of sevoflurane was greater in the striatum than in the cerebellum, resulting in increasing R_1_.

We used ketamine intramuscularly to induce anesthesia for the safety of animals. The decreasing concentration of ketamine could be a reason why we had to increase the sevoflurane concentration during the experimental session. In addition, ketamine might be another factor explaining the change of radioligand kinetics by having an effect on CBF [22]. A direct effect of ketamine on the dopamine system should also be considered. Some articles have reported that ketamine induces a decrease in [^11^C]raclopride binding [23, 24]. However, PET measurements in those studies started close to the time of ketamine infusion (just after or during ketamine infusion). This is different from the present study where PET started 2 h or more after ketamine administration. To rule out an effect of ketamine strictly, a study without initial administration of ketamine will be needed.

Besides anesthesia, a diurnal effect is another possible factor that might influence [^11^C]raclopride binding since several studies have suggested that the dopamine system may be affected by the circadian rhythm [25–27]. However, the interval between two PET measurements in this study was relatively short (2–3 h), compared to the interval in studies demonstrating a circadian rhythm, typically between morning and evening. Sevoflurane anesthesia is thus the most likely factor causing the demonstrated change of radioligand kinetics.

The present study suggests that these differences of radioligand kinetics were related to sevoflurane, and not caused by a change of specific binding. This means that NHP PET study with sevoflurane anesthesia is an appropriate method to evaluate the specific binding of radioligands. Ideally, the concentration of sevoflurane should be maintained throughout the experimental session. However, this maintenance of concentration is not always feasible since anesthesia levels have to be adjusted to the condition of the NHP. In studies where the changes of outcome measures are expected to be small, *e.g.*, occupancy study of agonist compound [28], it may be worth to increase the number of measurements to strengthen the statistical power. Moreover, if the concentration of sevoflurane changed drastically during the PET measurement, the result should be interpreted with caution.

In this study, neither [^15^O]H_2_O PET to measure the CBF nor arterial blood sampling to obtain an input function for estimation of the kinetic parameters including K_1_ was performed. The relation between sevoflurane concentration and CBF or K_1_ could accordingly not be evaluated. These issues should be solved in a future study.

In conclusion, we found a negative correlation between TTP in the kinetics of [^11^C]raclopride and the concentration of sevoflurane. However, this effect is not to be viewed as a major confounder since it did not propagate to a significant effect on the calculated BP_ND_ values. In NHP PET study with sevoflurane anesthesia, the concentration of sevoflurane should be maintained as much as possible in particular if small changes in radioligand binding are expected.
